# State Estimation for Early Feedback Responses in Reaching: Intramodal or Multimodal?

**DOI:** 10.3389/fnint.2017.00038

**Published:** 2017-12-19

**Authors:** Leonie Oostwoud Wijdenes, W. Pieter Medendorp

**Affiliations:** Donders Institute for Brain, Cognition and Behaviour, Radboud University, Nijmegen, Netherlands

**Keywords:** online movement control, multimodal integration, feedback control, state estimation, vestibular organ

## Abstract

Humans are highly skilled in controlling their reaching movements, making fast and task-dependent movement corrections to unforeseen perturbations. To guide these corrections, the neural control system requires a continuous, instantaneous estimate of the current state of the arm and body in the world. According to Optimal Feedback Control theory, this estimate is multimodal and constructed based on the integration of forward motor predictions and sensory feedback, such as proprioceptive, visual and vestibular information, modulated by context, and shaped by past experience. But how can a multimodal estimate drive fast movement corrections, given that the involved sensory modalities have different processing delays, different coordinate representations, and different noise levels? We develop the hypothesis that the earliest online movement corrections are based on multiple single modality state estimates rather than one combined multimodal estimate. We review studies that have investigated online multimodal integration for reach control and offer suggestions for experiments to test for the existence of intramodal state estimates. If proven true, the framework of Optimal Feedback Control needs to be extended with a stage of intramodal state estimation, serving to drive short-latency movement corrections.

## Optimality in perception and action

Perceiving and acting can be considered as two sides of the same coin. To serve the perception-action coupling, the sensory system has to estimate the state of the world (e.g., where and what are interesting objects) and body (e.g., where are my hands), while the motor system is concerned with prospective control based on these state estimates, i.e., generating the motor commands needed to acquire a particular task goal. Recent insights suggest that perception and action are not only intertwined at the computational level, following optimality principles (Todorov and Jordan, 2002; Körding and Wolpert, 2004; Shadmehr and Krakauer, 2008; Oostwoud Wijdenes et al., 2016), but also at the neural level (Cisek, 2006; Klein-Flugge and Bestmann, 2012; Grent-'t-Jong et al., 2014).

From a sensory perspective, optimality is defined as minimizing uncertainty about the state of the body and world by combining redundant information from different sensory modalities, weighting each signal in proportion to its reliability (van Beers et al., 1999; Ernst and Bülthoff, 2004; Körding and Wolpert, 2004). Indeed, psychophysical studies have shown that human perception is near-optimal when integrating visual-proprioceptive (van Beers et al., 1999), visual-haptic (Ernst and Banks, 2002), visual-auditory (Alais and Burr, 2004; Körding et al., 2007), or visual-vestibular information (Fetsch et al., 2009; ter Horst et al., 2015). In such studies, the typical approach was to estimate noise levels of the two sensory modalities in separate unimodal experiments, which were then used to predict perception in the bimodal case (but see Clemens et al., 2011 for a different approach). Also within the visual system, information available before and after an eye movement (Oostwoud Wijdenes et al., 2015), and current and remembered visual information appears to be integrated in an optimal manner (Brouwer and Knill, 2009; Atsma et al., 2016).

From a motor angle, optimality additionally includes factors other than variability. Next to controlling for task-relevant but not for task-irrelevant variability of the movement (Todorov and Jordan, 2002; Franklin and Wolpert, 2008; Scott, 2012), effort is also minimized, while accuracy and stability are maximized. These factors are weighted against movement reward, e.g., reaching the goal fast (Liu and Todorov, 2007). For any possible action, the brain needs to know the expected costs as well as the rewarding nature of the sensory states that it might achieve. This requires knowledge of body and world dynamics, called a forward internal model (Miall and Wolpert, 1996; Kawato, 1999; Shadmehr and Mussa-Ivaldi, 2012). Using this knowledge, the brain can compute the expected costs of particular movements, and subsequently select the most optimal movement.

By using the internal model, the brain also relates motor commands to their sensory consequences, which is mandatory to differentiate sensations that arise as a consequence of one's own movements from those that arise from changes in the environment (Cullen, 2004; Körding et al., 2007; Reichenbach et al., 2014). For example, the fact that we cannot tickle ourselves is evidence that the brain can predict (and thereby nullify) the consequences of its own action (Blakemore et al., 1998; Bays et al., 2006). In order to keep sensory predictions accurate, the forward model must be continuously calibrated to the actual dynamics of body and world, called motor adaptation (Wolpert et al., 1995; Shadmehr et al., 2010).

**Figure 1:**
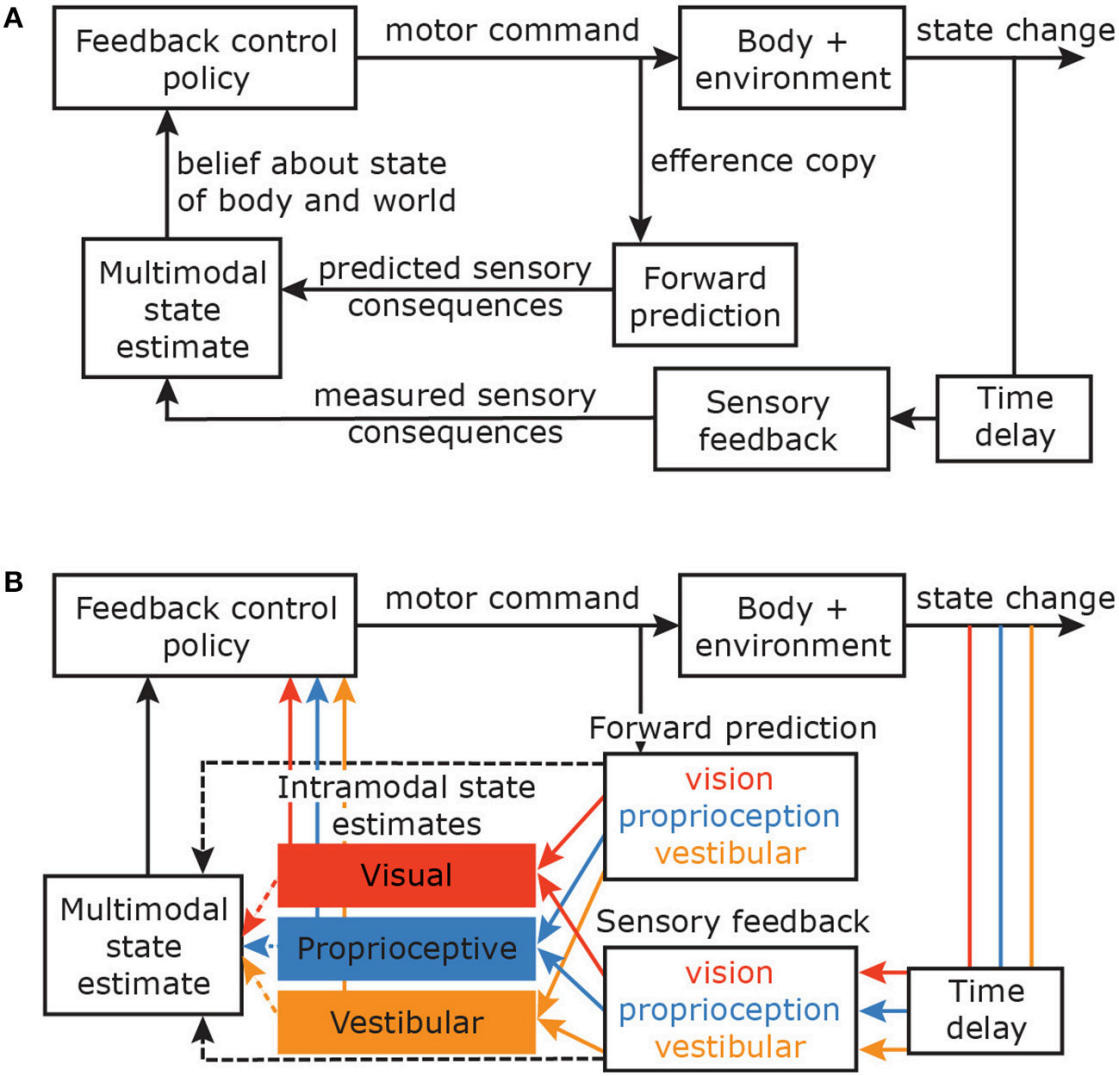
**(A)** Optimal Feedback Control framework (figure based on Shadmehr and Krakauer, 2008). Motor commands produce body movements. An efference copy of the commands is used to predict the sensory consequences of these commands. With some time delay, the sensory consequences of the actual movement are registered by different sensory modalities. The predicted and observed sensory consequences are combined to estimate the current state of the body in the world. This state estimate is fed into the feedback control policy and the feedback gains with which the system responds to perturbations are adapted accordingly (Franklin and Wolpert, 2008). This loop continues until the final desired state is reached. Although the brain does not use the mathematical tools of the OFC framework, we assume that it can describe the results of the actual processes. **(B)** OFC model with different sensory modalities and their time delays. It can be argued that the earliest stages of movement corrections are controlled via intramodal state estimates that are based on within-modality forward predictions and sensory feedback.

All these considerations imply a natural link between the sensory and motor systems, which is computationally captured by the Optimal Feedback Control (OFC) framework (Todorov and Jordan, 2002; Shadmehr and Krakauer, 2008) (Figure 1A). This framework proposes that the brain estimates the state of the body using a combination of sensory feedback from various modalities and forward predictions about the consequences of the commanded action, based on an internal model of the mapping between motor commands and their effect on the body in the world (Wolpert et al., 1995; Miall and Wolpert, 1996). This body state is then used to control action. However, it is still unclear how information from the forward prediction and the sensory feedback from different modalities are propagated to achieve a coherent multimodal state estimate.

In this article we focus on state estimation in the control of reaching movements. First, we discuss the major problems that the brain needs to solve in order to successfully integrate feedforward predictions and sensory feedback from multiple modalities, then we propose an extension to the OFC model that may help to solve these problems, and finally we suggest possible experiments to test the model extension.

## Continuous movement control

To accomplish reach tasks in an ever-changing world, it is essential to be able to control movements while they are executed, which we will call online control here. The online control of movements is arguably also the most demanding type of control in the link between perception and action because afferent sensory information is changing continuously and the time to make adjustments is limited. For online movement control, sensory information from multiple modalities has to be processed in a very short time frame in order to identify if the current course of movement will end on the desired location, and, if this is not the case, to make appropriate adjustments before the movement ends. For these adjustments to be successful, a reliable estimate of the current state of the body is essential.

The estimate of the current state of the arm can be examined by experimentally perturbing information about the current course of movement via one of the sensory organs and measuring the movement adjustments made in response to the imposed perturbation. Two sensory organs that provide proprioceptive information about the current state of the arm are muscle spindles and mechanoreceptors in the skin (Crevecoeur et al., 2017). After a mechanical perturbation, it takes about 50–100 ms before the hand shows task-dependent movement adjustments (for a review see Pruszynski and Scott, 2012). Within this time frame, movement adjustments depend on verbal instructions, and target and obstacle configurations (Hammond, 1956; Pruszynski et al., 2008; Nashed et al., 2014). Furthermore the gains of such task-dependent adjustments can be modulated throughout the movement (Mutha et al., 2008).

Visual information about the current state of the arm is provided by the eyes. It has been known for a long time that hand movements are under continuous visual control (Woodworth, 1899; Gielen et al., 1984; Pélisson et al., 1986). Even the earliest stages of a movement will be adjusted in response to visual target jumps (Georgopoulos et al., 1981; Van Sonderen and Denier van der Gon, 1991). Response latencies are in the order of 100-150 ms and not affected by movement stage (Gritsenko et al., 2009; Oostwoud Wijdenes et al., 2011; Sarlegna and Mutha, 2015).

Also perturbing visual feedback about current arm position will result in adjustments of the ongoing movement (Brenner and Smeets, 2003; Sarlegna et al., 2003; Saunders and Knill, 2004). In such experiments, participants reach to targets on a screen while seeing a cursor representing their hand position. Jumps of the cursor can probe movement corrections. Like responses to proprioceptive perturbations, adjustments in response to visual perturbations of the target and the cursor are dependent on the task at hand and evolve throughout the movement (Franklin and Wolpert, 2008; Gritsenko et al., 2009; Knill et al., 2011; Oostwoud Wijdenes et al., 2011, 2013; Dimitriou et al., 2013; Franklin et al., 2017) (for a review see Sarlegna and Mutha, 2015).

Furthermore, the vestibular system provides information about the current state of the body. Its sensory organs, the otoliths and semi-circular canals, detect linear acceleration and angular velocity of the head, respectively. It has been shown that vestibular information is also included in the continuous control of hand movements. Passive body rotations during reaches to remembered visual targets result in angular deviations of the hand that correspond to the perceived vestibular perturbation (Bresciani et al., 2002b; Reichenbach et al., 2016). Electrical stimulation over the mastoid processes that produces the illusion of a body rotation (galvanic vestibular stimulation) during the movement also results in online and task-dependent movement adjustments (Bresciani et al., 2002a; Keyser et al., 2017; Smith and Reynolds, 2017). Latencies of reach corrections in response to vestibular perturbations seem to be substantially longer than corrections in response to visual and proprioceptive perturbations, i.e., about 176–240 ms (Bresciani et al., 2002a; Moreau-Debord et al., 2014; Keyser et al., 2017). Preliminary data of experiments probing movement corrections with visual target jumps during passive body acceleration suggests that visuomotor feedback gains are modulated by vestibular input (Oostwoud Wijdenes and Medendorp, 2017). Thus, sophisticated hand movement adjustments are observed in response to perturbations of visual, proprioceptive and vestibular information.

Although the movement adjustment needed to correct for a perturbation of the world or the body can be the same, for example compare a 1 cm rightward target jump and a 1 cm leftward displacement of the representation of the hand (e.g., by means of a cursor jump), the underlying cause of the perturbation is different. For accurate perception and action, it is important that changes in the world are not attributed to changes in the body, and that changes in perception due to noise in the sensors are not attributed to changes in the world (Berniker and Kording, 2008). To help solving this agency problem for the visual system, there is a special binding mechanism that links visual and motor information about movement of the cursor (Reichenbach et al., 2014). For the vestibular system, neurons in the cerebellum are involved in the selective encoding of unexpected but not self-generated self motion (Brooks and Cullen, 2013).

## Challenges for online multisensory integration

Perturbation experiments can probe a single sensory modality, e.g., a visual target or cursor jump only perturbs the visual information, or multiple modalities e.g., a passive body rotation perturbs vestibular, proprioceptive and visual information. The current state of the arm can most reliably be estimated by combining information from different modalities, but this involves complex computations taking into account differences in noise properties, internal dynamics and intrinsic reference frames of the various sensors.

A challenge with integrating information from different sensory modalities for the online control of reaching is related to the processing of information in time (Cluff et al., 2015; Scott, 2016). Different sensors have different internal dynamics and involve different neural circuitries. Proprioceptive perturbations induced by a sudden mechanical displacement of the hand cause a stretch in the muscle spindles that almost immediately results in a stretch reflex via the spinal cord (Liddell and Sherrington, 1924). However, it takes 50–100 ms for the reach response to show task dependent modulations (for review see Pruszynski and Scott, 2012). Visual perturbations provoke a change to the input on the retina. Latencies to visual perturbations are somewhat longer than to proprioceptive perturbations, in the order of 100–150 ms (for review see Sarlegna and Mutha, 2015). Vestibular perturbations make the hair cells in the otolith organs or in the semicircular canals bend, which results in action potentials projecting to the vestibular nucleus and the cerebellum. Although for eye movements the first corrections in response to head motion take less than 15 ms (Sparks, 2002), latencies of hand movement corrections that take into account task demands are substantially longer, about 176–240 ms (Bresciani et al., 2002a; Moreau-Debord et al., 2014; Keyser et al., 2017). Thus if someone is thrown off balance during a reach, which perturbs the perceived position of the body proprioceptively, visually and vestibularly at the same time, movement corrections in response to this disturbance are manifested with different delays.

It is unknown which processing stage or stages cause these differences in behavioral delays. Next to sensor dynamics, an obvious difference between modalities is the way that information is encoded. Different sensors collect information about the current position of the hand in different coordinate systems. Proprioceptive afferent information is generally defined in a muscle-centered reference frame (Gardner and Costanzo, 1981). Visual information is initially defined in a retinotopic reference frame, and vestibular information in a head-centered reference frame (Raphan and Cohen, 2002). Later processing steps in the feedforward control of movement, such as reach planning, are carried out in multiple reference frames in large cortical networks (Beurze et al., 2006; McGuire and Sabes, 2009; Cappadocia et al., 2016), and also the hand state estimate is not defined in a single reference frame, but in a mixture of coordinate systems (Berniker et al., 2014). To arrive at these multimodal reference frames may require time-consuming neural coordinate transformations, although there are also modeling and empirical suggestions that the multilayer networks in the brain allow for automatic remapping of sensory inputs in multiple reference frames (Pouget et al., 2002; Azañón et al., 2010), perhaps mediated by neuronal oscillations (Buchholz et al., 2011, 2013; Fries, 2015).

## Intramodal state estimates

This raises the question of how the brain achieves fast and accurate online reach control. To deal with differences in delays within the Optimal Feedback Control framework, it has been proposed that the reliability of information from modalities for which it takes more time to evoke task dependent corrections should be reduced (Crevecoeur et al., 2016). Such an approach assumes that modality dependent delays originate from differences on the input side, thus in the sensor dynamics and conduction times to the CNS only. This assumption is in particular questionable when considering the vestibular system in state estimation for reaching movements. Why does it take 176–240 ms to evoke task dependent corrections of the arm? Vestibular ocular responses proceed much faster. Therefore it is unlikely that this delay reflects sensory conduction times only. In the following we will provide alternative reasoning on how the brain might deal with different sensory delays, which may also account for recently published findings.

Franklin et al. (2016) investigated if visual estimates of target and hand position are integrated in a common reference frame for the early online control of reaching. During forward reaching movements with a robotic manipulandum, the target of the reaching movement and the cursor that represented the unseen hand could both, independently, jump to a range of new locations. In half of the trials, the actual lateral position of the robot handle was fixed in order to measure corrective forces in response to the jumps. If the visual distance between the neural representations of hand and target location is the only direct input for the conversion from a spatial to a muscle-based reference frame (Bullock et al., 1998), changes in cursor and target position that result in the same visual distance should result in the same corrective forces (for example the force needed to correct for a 1 cm rightward target jump is the same as the force needed to correct for a 2 cm leftward hand-cursor jump in combination with a 1 cm left target jump). However, consistent with Brenner and Smeets (2003) who showed that simultaneous cursor and target jumps of the same size result in movement corrections, Franklin et al. (2016) found that the force depended on the relative contributions of target and cursor displacements rather than the absolute difference vector. Based on a multichannel model, they conclude that parallel, separate feedback loops within the visuomotor system control for early corrections to changes in visual target and visual hand location. If perturbations of different origin within the same modality are processed in separate channels, it seems reasonable to suggest that multimodal control for early corrections might be also processed in separate channels.

To ensure the short correction latencies that are essential to act promptly in unpredictable, dynamic environments, one could propose that the fastest stage of control is based on intramodal estimates (Figure 1B). Rather than integrating information from multiple modalities, feedforward and feedback information of individual modalities are integrated to estimate the state of the hand based on a single modality. Within this notion, different modalities project via separate channels to the feedback control policy, or taking it a step further, there even might be channel-specific control policies. This type of control circumvents the spatial and temporal challenges related to integrating information from different sensory modalities, and might explain the different latencies that are found to compensate for changes in different modalities. However, such a mechanism lacks in reliability: integrating information from multiple modalities, if congruent, will make the estimate more reliable. Thus a multimodal state estimate probably should control later stages of the movement. Integration for multimodal state estimation may hence be based on intramodal state estimates or on direct feedforward and feedback input from the different sensory organs.

To our knowledge, only few studies have investigated multimodal integration for the online control of reaching by independently perturbing more than one sensory modality. Mutha et al. (2008) investigated the integration of visual and proprioceptive information. They asked participants to make 30° elbow extension movements and on some trials the target jumped 15° toward or away from the start position at movement onset. In addition, 100 ms after the visual perturbation, they mechanically pushed the arm closer to the target, or away from the target. They found that the response to the proprioceptive perturbation was affected by the visual perturbation. If the target jump and the mechanical perturbation were in the same direction, the force that was produced to correct for the perturbations was lower than if the visual and proprioceptive perturbation were in opposite directions. This suggests that the multimodal state estimate is updated quickly and accurately. However, in a second experiment they varied the amplitude of the visual perturbation and were unable to find amplitude related modulations in the corrections for proprioceptive perturbations. This non-linearity in the responses might suggest that early components of the responses may be modulated by separate intramodal state estimates rather than one multimodal estimate, because a multimodal estimate should be optimally tuned to the task.

Crevecoeur et al. (2016) also investigated the integration of visual and proprioceptive information for the online control of movement. Specifically, they asked if the nervous system integrates visual and proprioceptive information based on the sensory reliability, as is typically the case for static perception (van Beers et al., 1999; Ernst and Banks, 2002), or whether it also takes into account the differences in time delays between modalities. They argue that, because it takes longer for visual than for proprioceptive perturbations to affect the hand movement, visual feedback is more corrupted by noise and therefore the brain should discount visual information. Participants were asked to stabilize their finger on a dot. After a short delay their arm was either mechanically perturbed without visual feedback, or the hand-cursor was visually perturbed along a trajectory corresponding to the path of a mechanically perturbed arm, or their arm was mechanically perturbed with visual feedback of the cursor (mechanical + visual perturbation). For mechanical perturbations, participants were instructed to quickly move their hand back to the start dot while looking at their unseen finger. For visual perturbations they were instructed to visually track the cursor. Inventively, Crevecoeur et al. (2016) took gaze as a proxy for the state estimate of the hand location. They found that saccadic latencies were shorter in response to the mechanical and the mechanical + visual perturbations than to the visual perturbation alone. This result supports a multisensory integration model that takes into account the differences in time delays between visual and proprioceptive information. Alternatively, the similarities between mechanical and mechanical + visual response latencies could be explained by early intramodal feedback control, because in that case one would expect an adjustment to start as soon as one of the modalities, in this case proprioception, detects that movement corrections are needed.

Finally, Crevecoeur et al. (2017) investigated the integration of information from skin mechanoreceptors and muscle spindles. Both sources provide information that supports the control of finger movements. Crevecoeur et al. (2017) asked how information from these two sensory modalities is integrated. Participants were asked to touch a surface that could move underneath their index finger. When they perceived surface motion they were asked to push onto the surface to prevent it from slipping. In a two-by-two design they did or did not restrain actual movement of the finger, and the mechanoreceptors of the finger were or were not anesthetized. They found that the initial response to the surface motion at a latency of ~60 ms was modulated by muscle spindle feedback only, since anesthetizing the mechanoreceptors did not affect the response. It took ~90 ms for mechanoreceptor feedback to start contributing to the response. After this time, it seems that mainly finger motion and to a lesser extent strain affects the movement correction, which is not directly what optimal integration would predict. They concluded that the two sensors operate in partially distinct sensorimotor circuits, congruent with the proposal that intramodal state estimates drive short-latency movement corrections.

## Testing intramodal state estimation

It needs to be tested if the idea of intramodal state estimates for reach control holds water. This is not straightforward to do, because it is difficult to continuously track the state estimate during hand motion (Crevecoeur et al., 2016). Also, over time predictions for models with and without intramodal estimates converge because at longer delays the input for intra- and multimodal state estimation is the same. The critical moment where intramodal estimates give other predictions than a multimodal estimate is in the first couple of 100 ms after the perturbation. During this time, corrections would not be based on a weighted combination of all modalities determined by their reliability, but they would only depend on the modality that first detects the perturbation.

One way to test if the brain constructs intramodal estimates might be to alter the state estimate via one sensory modality and test how state estimates are updated via other modalities (Bernier et al., 2007). For example, one could teach participants a contraction or expansion of the visual consequences of their movements (Hayashi et al., 2016), or teach them to reach in a pulling or pushing force field. Throughout learning, on reaches to the straight-ahead target, one could probe the state estimate with visual, proprioceptive, and vestibular perturbations. If the brain uses intramodal state estimates, the earliest movement corrections for perturbations of the trained modality should result in a response congruent with the new sensorimotor mapping, while the earliest responses of other modalities should not reflect the new mapping until the multimodal state estimate is updated. If responses are the same irrespective of whether the modality was trained directly or indirectly, this would suggest that that even the fastest stage of control is based on a single multimodal state estimate.

So far, the majority of studies investigating online movement corrections used visual and proprioceptive perturbation paradigms. The vestibular system is known to play an important role in sensorimotor control as well (for review see Medendorp and Selen, 2017). We propose to also exploit this modality more extensively when investigating multimodal integration for online control. For example, by using Galvanic Vestibular Stimulation (GVS) (Fitzpatrick and Day, 2004) it is possible to perturb the vestibular input without mechanically affecting hand position – all changes in hand position are related to vestibular feedback responses (Keyser et al., 2017). This enables one to zoom in on the effect of the perturbation without the need to control for corrections due to stretching the arm muscles.

## Concluding remarks

In conclusion, the online control of reaching movements in the fast and fine-tuned fashion that humans typically display puts high demands on reference frame transformations and requires internal knowledge about conduction time delays of different sensors. Here we considered the novel idea that in light of the speed with which corrections are observed, the earliest adjustments to ongoing movements may be based on intramodal state estimates. Experimental and modeling studies should investigate if this would be a valuable extension to the Optimal Feedback Control framework. Although we have focused on reaching movements here, the framework extends to other types of continuously controlled movements of the arm and hand, such as grasping (Voudouris et al., 2013), as well as those of the leg (Potocanac et al., 2014).

## Author contributions

All authors listed, have made substantial, direct and intellectual contribution to the work, and approved it for publication. LOW: conceptualization, writing—original draft preparation, writing—review and editing. WPM: conceptualization, funding acquisition, supervision, writing—review and editing.

### Conflict of interest statement

The authors declare that the research was conducted in the absence of any commercial or financial relationships that could be construed as a potential conflict of interest.
